# A Manual of Otology

**Published:** 1901-02

**Authors:** 


A MANUAL OF OTOLOGY. By GORHAM BACON, A B., M.D., Professor of Otology in Cornell University Medical College, New York; Aural Surgeon, New York Eye and Ear Infirmary. With an introductory chapter by Clarence John Blake, M. D., Professor of Otology in Harvard University. Second edition, revised and enlarged With 114 illustrations and three colored plates. 12mo. pp. 422. Philadelphia: Lea Brothers & Co. 1900. [Price, $2.00]

In less than two years a second edition of Bacon's Manual of Otology has been called for, and the response has been met by a searching revision throughout. The number of illustrations has been increased and additions have been made to the text to the extent of some twenty-five pages. The new matter thus incorporated includes a more extended consideration of the Schwartze-Stacke operation, and the use of the normal saline solution in intravenous injections. Dr. Blake, who has given his special favor to the work, has also enlarged his introduction.

The work is exceedingly practical, and is a most complete epitomisation of modern otology. We again commend it unqualifiedly, especially to the student. A. A. H.

